# Fishing Into the MicroRNA Transcriptome

**DOI:** 10.3389/fgene.2018.00088

**Published:** 2018-03-19

**Authors:** Marcos E. Herkenhoff, Arthur C. Oliveira, Pedro G. Nachtigall, Juliana M. Costa, Vinicius F. Campos, Alexandre W. S. Hilsdorf, Danillo Pinhal

**Affiliations:** ^1^Laboratory of Genomics and Molecular Evolution, Department of Genetics, Institute of Biosciences of Botucatu, Sao Paulo State University, Botucatu, Brazil; ^2^Laboratory of Structural Genomics (GenEstrut), Graduate Program of Biotechnology, Technology Developmental Center, Federal University of Pelotas, Pelotas, Brazil; ^3^Unit of Biotechnology, University of Mogi das Cruzes, Mogi das Cruzes, Brazil

**Keywords:** aquaculture, farm animals, teleost fish, gene expression, microRNAs

## Abstract

In the last decade, several studies have been focused on revealing the microRNA (miRNA) repertoire and determining their functions in farm animals such as poultry, pigs, cattle, and fish. These small non-protein coding RNA molecules (18–25 nucleotides) are capable of controlling gene expression by binding to messenger RNA (mRNA) targets, thus interfering in the final protein output. MiRNAs have been recognized as the main regulators of biological features of economic interest, including body growth, muscle development, fat deposition, and immunology, among other highly valuable traits, in aquatic livestock. Currently, the miRNA repertoire of some farmed fish species has been identified and characterized, bringing insights about miRNA functions, and novel perspectives for improving health and productivity. In this review, we summarize the current advances in miRNA research by examining available data on Neotropical and other key species exploited by fisheries and in aquaculture worldwide and discuss how future studies on Neotropical fish could benefit from this knowledge. We also make a horizontal comparison of major results and discuss forefront strategies for miRNA manipulation in aquaculture focusing on forward-looking ideas for forthcoming research.

## Introduction

MicroRNAs (miRNAs) are a class of small (17–22 nucleotides), non-coding RNAs that inhibit gene expression post-transcriptionally by pairing with complementary sequences in their target mRNA (Box 1). These small regulatory molecules are present in the genome of animals, plants, and even some viruses (Lee et al., 1993; Bartel, 2004; Kim et al., 2009; Xia et al., 2011). The first miRNAs, lin-4 and let-7, were both discovered in *Caenorhabditis elegans* (Lee et al., 1993; Wightman et al., 1993; Reinhart et al., 2000) and have subsequently been found to correspond to a novel and extensive class of small non-coding RNAs (Lagos-Quintana et al., 2001; Lau et al., 2001; Lee and Ambros, 2001).

Box 1 **miRNA biogenesis and action.**

The miRNA genes are firstly transcribed in the nucleus by RNA polymerase II to generate primary miRNAs (pri-miRNAs). In the canonical pathway, the pri-miRNAs bend into hairpins and are processed by the RNase III Drosha to form precursor miRNAs (pre-miRNAs) with around 70 nt, characterized by the stem-loop structure (Lee et al., 2003; Kim, 2005). The pre-miRNA is transported from the nucleus to cytoplasm by Exportin-5 and is recognized and cleaved by the RNase III Dicer to form a double-stranded RNA with 22 nt (Hutvágner et al., 2001; Lund et al., 2004). The double strands unbind and one of the strands enters in the RNA-induced silencing complex (RISC), becoming a mature miRNA that can exercise its gene silencing function, whereas the other strand is generally degraded or, in some cases, can also form an RISC (Khvorova et al., 2003).

In addition to the canonical pathway, there are alternative pathways that can promote miRNA biogenesis. One of them is the “Drosha/DGCR8-independent pathway” (Ha and Kim, 2014). In this process, the pre-miRNA (also called mirtron) comprises the whole intronic region of a gene. Thus, when the spliceosome cleaves the transcript, the pre-miRNA is released, and its biogenesis follows the standard pathways. The “TUTase-dependent pathway” (Ha and Kim, 2014) is another type of alternative processing of pri-miRNAs. In this pathway, TUT family proteins binds to pre-miRNAs with only one nucleotide overhang in the 3' region and adds a uridine in its 3' end, promoting the two-nucleotide overhang, important for the correct miRNA maturation. Another type of non-canonical pathway is the “Dicer-independent pathway” (Ha and Kim, 2014). During this process, AGO2 proteins bind directly to the pre-miRNA, and along with PARN ribonucleases, promote the cleavage and final maturation of the miRNA.

All the aforementioned pathways lead to miRNA maturation and subsequent miRNA activity occurs through miRNA-RISC complex binding mainly to the 3′ UTR region of target mRNA, inhibiting the expression or degrading the mRNA transcript (Lee and Dutta, 2009). Besides the 3' UTR region, non-canonical miRNA-to-target interactions were described to occur in binding sites located inside exons (Reczko et al., 2012; Hausser et al., 2013) orin the 5' UTR region (Devlin et al., 2010; Zhou and Rigoutsos, 2014). It has been estimated that each miRNA may modulate hundreds of messenger RNAs and a single messenger RNA may have its stability or translation controlled by several miRNAs (Doench and Sharp, 2004; Brenneck et al., 2005; Lim et al., 2005).

Since then, numerous miRNAs have been identified and quantified in several organisms, primarily as a result of numerous enhancements in high throughput sequencing technologies, bioinformatics computer programs and experimental methods (Guerra-Assumpção and Enright, 2012). Together, these approaches have enabled large-scale analyses, thereby facilitating the discovery of species-specific and novel miRNAs, even those with reduced expression, in many organisms belonging to diverse taxa (Berezikov, 2011).

Owing to the key involvement of miRNAs in the regulation of growth, metabolism, and homeostasis, among hundreds of other functions, diverse studies over the last decade have sought to identify genuine miRNA-to-target interactions in farm animals, such as poultry, pigs, cattle (reviewed by Wang et al., 2013), and fish (Rasal et al., 2016), to maximize production (Box 2). In addition, several patents have been approved in various countries for the commercial exploration of miRNAs, e.g., a US patent (US 2006/0246491 A1) using miRNAs to regulate muscle cell growth.

Box 2 **Finding miRNA putative targets.**

Searching for a true miRNA-mRNA interaction is a difficult task. Despite the availability of several computational target prediction tools, their results are generally inconsistent and often return variable sets of possible miRNA targets. This happens mainly because the rules governing miRNA target recognition are not completely understood and may vary for each miRNA-target interaction (Ritchie and Rasko, 2014). Moreover, several popular tools such as DIANNA-microT (Maragkakis et al., 2009), miRanda-miRSVR (Betel et al., 2010), and miRWalk (Dweep et al., 2011) were designed exclusively for predicting miRNAs in mammals. Other tools, such as TargetScan (Garcia et al., 2011), miRanda (Enright et al., 2003), PITA (Kertesz et al., 2007), and RNA22 (Miranda et al., 2006) algorithms are frequently used as target prediction tools for animal miRNAs (Witkos et al., 2011; Reyes-Herrera and Ficarra, 2012; Dweep et al., 2013; Peterson et al., 2014).

Once there are several options of target prediction tools available in the literature, researchers often look up for which tool or combination of tools allows for the best quality results. Oliveira et al. (2017) shows that the union of the results provided by TargetScan, miRanda and RNA22 provide a better performance (balance of sensitivity and specificity) than any of these tools alone or other combination of them. However, it is important to keep in mind that any target prediction tool or combinatory use may still return false-positive results.

Since the target prediction tools yet lack consistency, once putative targets have been selected, validation experiments must be performed to confirm if the interaction is genuine. The majority of strategies for miRNA-to-target validation rely on the overexpression and/or knockdown of the miRNA of interest and corresponding surveillance of the effect at the mRNA/protein levels of predicted target genes. To evaluate the mRNA and/or protein expression profiles associated with the manipulation of a specific miRNA, several functional analyses have been widely applied (Table 1).

**Table 1:** Summary of studies on miRNAs of Neotropical and other fish species with relevance on aquaculture.

**Group**	**Species**	**Tissue**	**Methodology**	**References**
Neotropical	Tambaqui	Liver and skin	Illumina sequencing technology and functional annotation	Gomes et al., 2017
	Pacu	Skeletal muscle	Real-time PCR	Paula et al., 2017
	Midas cichlids	Not informed by authors	Target prediction and Illumina sequencing technology	Franchini et al., 2016
Others	Nile tilapia	embryo	RNA-seq high-throughput sequencing and miRNA target prediction analysis	Eshel et al., 2014
		Gonads	Solexa sequencing with real-time PCR expression, miRNA target prediction analysis and miRNA pathways analysis	Wang et al., 2016
			Solexa sequencing with real-time PCR expression	Xiao et al., 2014
		Kidney	Real-time PCR and luciferase reporter assay	Yan et al., 2012b,c; Zhao et al., 2016
		Skeletal muscle	Prediction of MyoD-binding miRNAs, real-time PCR and luciferase reporter assay	Khvorova et al., 2003; Yan et al., 2013b
			Microarray and luciferase reporter assay	Kim et al., 2006
			Silencing of miR-206 *in vivo* using the antagomir method	Yan et al., 2013b
	Atlantic Salmon	Blood	RNA-seq high-throughput sequencing	Kure et al., 2013
	Rainbow trout	Larvae	Real-time PCR	Ramachandra et al., 2008
		Liver	Prediction of miRNA targer gene and real-time PCR	Mennigen et al., 2012
		Muscle	RNA-seq high-throughput sequencing and target prediction	Salem et al., 2010
	Grass carp	Blood	Solexa sequencing technology, real-time PCR, directional cloning and induction of expression of three immune factors in vitro.	Xu et al., 2016
		Embryo	Real-time PCR	Xu et al., 2014
		Spleen	Solexa sequencing technology, real-time PCR, target prediction and integration with mRNA expression and luciferase report system	Xu et al., 2015

Here, we review the available data on miRNA identification and functional characterization in the most important globally farmed fish species, with an emphasis on Neotropical fish, and discuss the current challenges and future directions for the use of miRNAs in aquaculture.

## MiRNAs in animal breeding

**Figure 1:**
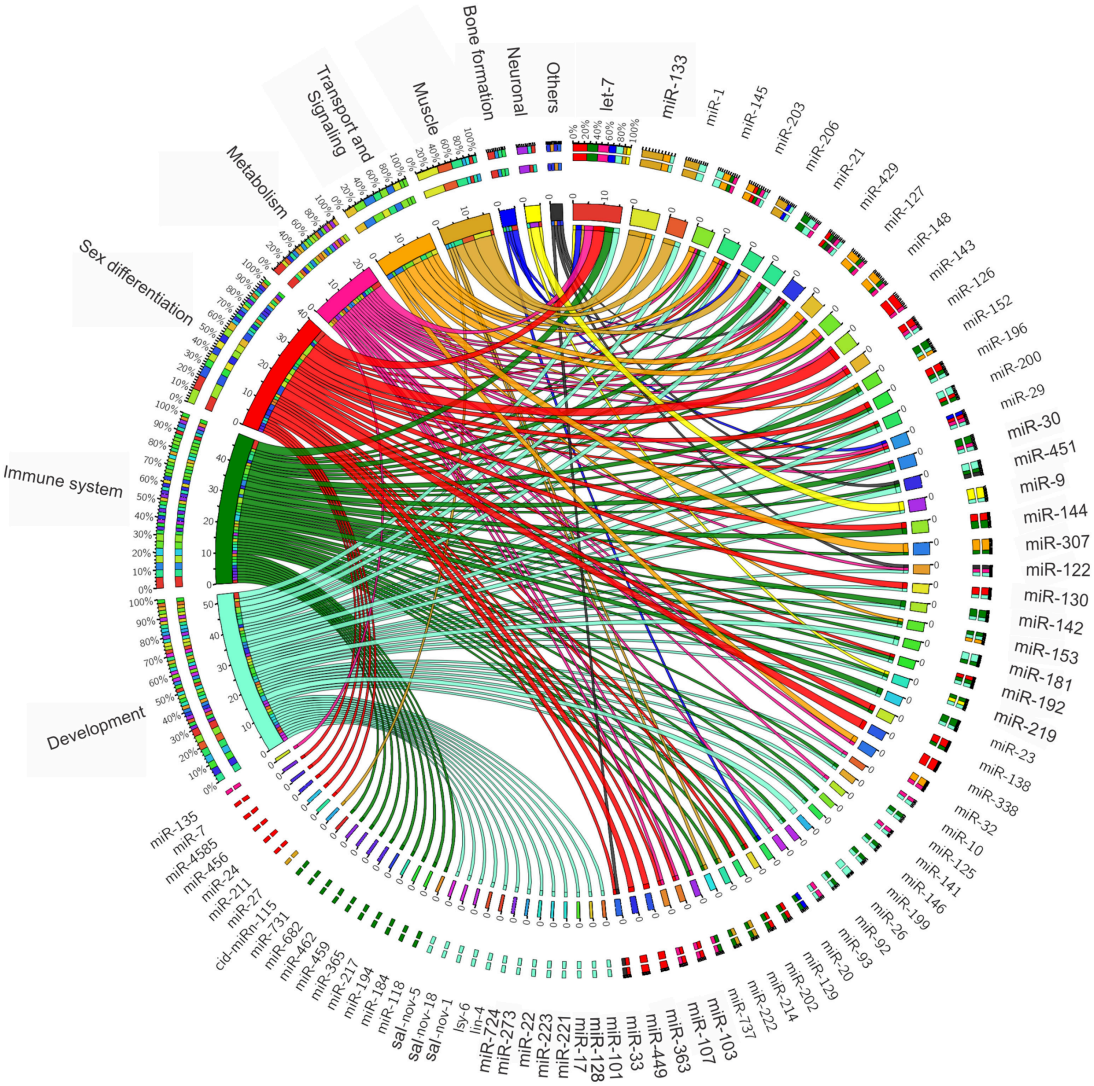
MicroRNAs functional association to variable biological contexts of relevance to farmed fish production.

Since miRNAs are key elements in gene regulation, they have been the focus of studies on improving the health and productivity of farm animal species. Diverse studies have been conducted in cattle (Townley-Tilson et al., 2010; Kozomara and Griffiths-Jones, 2011; Li H. et al., 2011; Miretti et al., 2011), pigs (Cho et al., 2010; Cirera et al., 2010; Xie et al., 2010; Li G. et al., 2011; Chen et al., 2012), and birds (Hicks et al., 2008, 2009; Li T. et al., 2011; Yao et al., 2011; Wang et al., 2012b). These studies have reported that miRNAs play an important role in a wide range of biological pathways, such as growth, metabolism, immunology, muscle development, and fat deposition (Figure 1). Therefore, these small RNAs are of great value in animal breeding, steering research toward the development of new solutions for current and emerging threats to the health and welfare of farm animal species.

Reports aimed at the identification and functional analysis of miRNAs in fish have increased recently, allowing for the characterization of the miRNA repertoire in Neotropical fish and in other prominent fish species in the aquaculture industry worldwide (Table 1).

## A review of miRNAs in farmed fishes

### Neotropical fish

Neotropical fish encompass 50% of all freshwater species that are currently identified, which includes ~13,000 characterized species (Reis et al., 2003; Bertollo et al., 2017). These data highlight the great biodiversity of this group of fishes and, particularly, of those of the Neotropical region. However, despite the growing interest in the study of Neotropical fishes, resources describing miRNA diversity and function are still very limited. Below, we provide a picture of the current knowledge of miRNAs of Neotropical species.

#### Tambaqui (*Colossoma macropomum*, characidae)

The black pacu or tambaqui (*Colossoma macropomum*) can reach up to 1 m in length and ~30 kg in weight and is a main resource of aquaculture and fisheries along the Amazon (Goulding and Carvalho, 1982; IBAMA, 2000). Gomes et al. (2017) characterized the miRNA expression profiles of liver and skin tissues of the Amazon tambaqui. They identified 279 conserved miRNAs in this species, with 257 from the liver and 272 from the skin and with several miRNAs expressed in both tissues. A functional enrichment analysis was performed for the targets of the 10 most highly expressed miRNAs of each tissue, revealing enriched biological pathways associated with metabolism, cell proliferation, calcium/ion transport and carbohydrate kinase activity in the liver as well as regulation of transcription and metabolic processes in the skin.

miR-122, which is highly expressed in the liver of tambaqui (Gomes et al., 2017), is conserved among vertebrate species and is known to be integrated with the regulation of cholesterol metabolism. Moreover, studies in rainbow trout corroborate the influence of miR-122 in liver metabolism (Mennigen et al., 2012, 2013). Thus, miR-122 is an interesting regulatory gene that requires further study regarding its impact on fish metabolism and, consequently, in aquaculture production.

#### Pacu (*Piaractus mesopotamicus*, characidae)

The pacu (*Piaractus mesopotamicus*), which is characterized by hardiness, fast growth, adaptation to artificial feeding and flavorsome meat (Castagnolli and Cyrino, 1986), is a valuable resource in Brazilian aquaculture (Urbinati and Gonçalves, 2005). Duran et al. (2015) and Paula et al. (2017) studied the role of skeletal muscle miRNAs in this Neotropical fish during development and under food restriction, respectively. Duran et al. (2015) analyzed the impact of the miRNA-target interactions of miR-1/*hdac4*, miR-133-a/b/*srf*, miR-206/*pax7*, and miR-499/*sox6* in fast- and slow-twitch skeletal muscles during growth. They found that miR-1 and miR-206 may promote myoblast differentiation in fast- and slow-twitch muscles in adult individuals, while miR-133a/b acts earlier, promoting myoblast proliferation in juveniles. Finally, miR-499 may promote differentiation of slow-twitch muscle fibers, which corroborates previous findings in Nile tilapia (Nachtigall et al., 2015). Paula et al. (2017) showed that a short period of food restriction significantly increased the expression of miR-1, miR-206, miR-199, and miR-23a in fast muscle and significantly decreased the expression of miR-1 and miR-206 in slow muscle, while their targets (*IGF-1* for miR-1, miR-206, and miR-199; *mTOR* for miR-199; and *MFbx* and *PGC1a* for miR-23a) exhibited negatively correlated expression profiles. Altogether, these data suggest that miRNAs may have a large impact on muscle plasticity and recovery after periods of food restriction.

MiR-1, miR-133a, miR-133b, miR-206, and miR-499 have been shown to be involved in the control of genes related to myoblast proliferation and differentiation. The same miRNAs were also found in the skeletal muscle of other farmed animals and exhibited a highly conserved genomic context (i.e., structural features) among fish species (Nachtigall et al., 2014). These findings underpin the putative applicability of these muscle miRNAs as valuable biomarkers in Neotropical fish breeding. Additional experiments focusing on the characterization of SNPs in miRNAs and target genes from distinct populations exhibiting variable phenotypic traits, such as increased muscle growth and body mass, are required.

#### Midas cichlids (*Amphilophus* spp., cichlidae)

The Midas cichlids are Neotropical fishes from Nicaraguan crater lakes. These species are considered to be an excellent model for studying speciation due to their fast parallel sympatric speciation. In contrast to other research that focused on miRNA expression and function, Franchini et al. (2016) studied the role of miRNAs in the diversification of five species of the Midas cichlid lineage (*Amphilophus* spp.).

Interestingly, by examining the putative effects of mutations of 3'UTR miRNA binding sites on the phenotypic diversification and evolution of these cichlids, the authors found that the species from Lake Apoyo (*A. astorquii* and *A. zaliosus*) have undergone less natural selection compared to species from Lake Nicaragua and Lake Managua (*A. citrinellus*) and Lake Xiloà (*A. amarillo* and *A. sagittae*). The impact of variable natural selection in the genome allowed novel mutations in binding sites to accumulate and the regulatory networks to evolve faster in the latter species, thus contributing to novel miRNA-target interactions associated with the phenotypic diversification of Midas cichlids (Franchini et al., 2016). These findings corroborate previous data regarding the “evolutionary race” between miRNAs and targets that have been proposed to influence the evolution of African cichlids (Loh et al., 2011; Brawand et al., 2014), thus indicating the pervasiveness of mutual molecular mechanisms of miRNA functional diversification in fish.

From an aquaculture perspective, such miRNA-target evolutionary mechanisms have selected adaptive traits for a specific environment, which can be tracked to identify economically valuable phenotypes. Moreover, this study reinforces the importance of examining population polymorphisms of both miRNAs and targets while performing fish genetic breeding programs.

### Other key fish species

Studies of miRNAs in Neotropical fishes are very recent, still providing a limited source of data regarding the impact of these molecules in Neotropical fish biology. Thus, in this section, we describe advances based on investigations of miRNA repertoires from other four fish species of relevance in aquaculture and fisheries worldwide and discuss the putative transferability of the acquired knowledge to Neotropical fishes.

#### Nile tilapia (*Oreochromis niloticus*, cichlidae)

Nile tilapia is one of the most commonly farmed species in freshwater aquaculture worldwide. A fast growth rate and adaptation to a wide range of culture conditions are the attributes responsible for successful fish farming (Yue et al., 2016).

Several studies aimed at miRNA characterization have been carried out in this model species. Using bioinformatics, Loh et al. (2011) analyzed the evolution of miRNAs and their target genes in cichlid fishes from Lake Malawi (East Africa) and detected 100 cichlid miRNAs, including those from tilapia, that are highly conserved in metazoan genomes. Brawand et al. (2014), who also studied cichlid miRNAs, described diverse loci in tilapia comprising 7 novel miRNAs. Yan et al. (2012a), using small RNA cloning and Sanger sequencing, discovered 25 conserved miRNAs in tilapia skeletal muscle.1'. Huang et al. (2012) employed next-generation sequencing (NGS) in muscle tissue to explore the complete miRNA transcriptome and identified the expression of 184 known mature miRNA sequences.

The functional roles of miRNAs have also evaluated under variable contexts. Several studies have investigated muscle development because muscle constitutes the major edible part of fish and is therefore an economically important trait. Many miRNAs have been proven to participate in the regulation of muscle growth by controlling the genes involved in hyperplasia and hypertrophy (Lagos-Quintana et al., 2002). Studies that focused on the initial embryonic development have also clarified miRNA targets involved in the tightly regulated initial steps of body formation (Giusti et al., 2016).

In tilapia, Yan et al. (2012a) and Nachtigall et al. (2015) showed that miR-1, miR-133a, and miR-206 have similar expression patterns in adult males and females and may assist each other to accurately control the development of skeletal muscles, although they perform distinct biological functions. MiR-1 is responsible for repressing the expression of histone deacetylase 4 (HDAC4), which is a negative regulator of cellular differentiation and thus promotes myocyte differentiation. MiR-1 also blocks a repressor of the MEF2 (myocyte enhancer factor-2) transcription factor. MiR-133a promotes, in part, myocyte proliferation by repressing serum response factor (SRF) (Chen et al., 2006), whereas miR-206 plays an important role in regulating the differentiation of C2C12 myoblasts *in vitro* (Kim et al., 2006). In addition, miR-206 loss of function *in vivo* was shown to significantly improve tilapia growth performance by targeting insulin-like growth factor-1 (IGF-1) (Yan et al., 2013b). IGF-1 is known to play a central role in a complex system that regulates growth, differentiation, and reproduction by selectively promoting mitogenesis and cell differentiation and inhibiting apoptosis (Jones and Clemmons, 1995; Reinecke and Collet, 1998). In many fish species, IGF-1 blood or tissue levels positively correlate with dietary protein levels and body growth rate (Beckman et al., 2004; Carnevali et al., 2006). Therefore, miR-206 could affect tilapia growth by modulating IGF-1 gene expression levels (Yan et al., 2014). Similarly, miR-203b has been shown to promote myogenesis (hyperplastic growth) by targeting MyoD (Yan et al., 2013a), a key protein that initiates the cascade of regulatory events during muscle differentiation. These authors showed that blocking miR-203b results in a significant increase in MyoD expression. In comparison to other approaches, however, using metabolic alterations, Wang D. et al. (2017) showed that miR-223-3p promotes upregulation of pou1f1, a key transcription factor related to somatic growth in Nile tilapia. Pou1f1 binds to the promoter region and transactivates the expression of growth hormone (GH), prolactin (PRL), and somatolactin (SL) in teleost fish. Furthermore, Qiang et al. (2017c) showed that miR-29a antagomir treatment *in vivo* resulted in stearoyl-CoA desaturase (SCD) upregulation, which plays a role in hepatic lipid metabolism regulation (Dobrzyn and Ntambi, 2004; Ntambi and Miyazaki, 2004).

Other studies have revealed miRNAs regulating metabolic pathways that could indirectly enhance production. For example, there is growing concern about the genetic improvement of salt tolerance in Nile tilapia, which may provide advantages, such as adaptation to certain environmental conditions and higher oxidation. Tilapia are euryhaline fish, and most species can live in a wide range of salinities from freshwater to seawater and therefore are a suitable model organism for studies on ionic and osmotic acclimation in euryhaline teleosts (Deane and Woo, 2004; Wang et al., 2009). For example, miR-30c, a kidney-enriched miRNA, was shown to regulate salt tolerance because its loss of function caused fish to be unable to respond to osmotic stress (Yan et al., 2012b, 2013b). Moreover, osmotic stress transcription factor 1 (OSTF1) was shown to be potentially regulated by miR-429 (Yan et al., 2012c). Recently, Zhao et al. (2016) showed that miR-21 is abundantly expressed and its action modulates alkalinity stress by upregulating VEGFB and VEGFC expression *in vivo* and *in vitro*, which are responsible for regulating alkalinity tolerance. Therefore, miR-21, miR-30c, and miR-429 may be important markers for tilapia and also for other commercial species. Nevertheless, Qiang et al. (2017d) concluded that miR-122 plays an important role in regulating the stress response in the lineage of Nile tilapia liver exposed to cadmium.

Another relevant topic for Nile tilapia production involves sex determination and differentiation, because males grow faster and larger than the female. Therefore, several studies have sought to uncover the molecular mechanisms of sex-determination regulated by miRNAs (Eshel et al., 2014; Xiao et al., 2014).

In the study by Xiao et al. (2014), gonads (testes and ovaries) were screened using high-throughput sequencing, and the data showed distinct miRNA expression signatures. In addition, Nile tilapia testes and ovaries displayed miR-181a, miR-181a-5p, miR-143, and miR-143-3p as the most abundant miRNAs. By contrast, the miR-29 and miR-129 families showed significantly increased expression in ovaries compared to testes (Xiao et al., 2014). In humans, the high expression of miR-129 in ovaries is associated with the control of cell growth and differentiation in the final process of ovary maturation through downregulation of its target mRNAs, whereas miR-29 expression levels, which progressively increase throughout oogenesis, may also be important (Sirotkin et al., 2009). In tilapia testes, the most abundantly expressed miRNA families are miR-33a, miR-132, miR-135b, and miR-212 (Xiao et al., 2014). In mammals, miR-212/132 expression is necessary for the development and function of neurons. Furthermore, both miRNAs are associated with mammary gland development by downregulating the matrix metalloproteinase 9 (MMP-9), which is an activator of TGFβ and is involved in cell proliferation, differentiation, and apoptosis (Kubiczkova et al., 2012). miR-33a controls fatty acid regulation in mammals by repressing insulin receptor substrate 2 (Dávalos et al., 2011), and its high expression in tilapia testes may contribute to testes maturation via regulation of the insulin signaling pathway (Xiao et al., 2014).

Eshel et al. (2014) compared miRNA expression in tilapia gonads and found nine sexually dimorphic expressed miRNAs; they found a single upregulated miRNA in male embryos (miR-4585) with a perfect inverse correlation in expression pattern with its six target genes, cr/20β-hsd, psmb8, rtn4ip1, casp8, atp5g3, and a non-annotated gene (downregulated in males). cr/20β-hsd is known to be part of the oxidoreductase pathway for oocyte maturation preceding the enzymatic activity of cyp19 (cytochrome P450 aromatase) (Senthilkumaran et al., 2004), and cyp19a1a is proposed to be the major gene for female determination in zebrafish (*Danio rerio*) (Rodríguez-Marí et al., 2010). MiR-4585 is upregulated in male embryos at 2- and 5-days post-fertilization (dpf) and decreases at 9 dpf, which indicates the significance of this miRNA in males soon after fertilization (Eshel et al., 2014). Therefore, miR-4585 could possibly be manipulated for sex reversal in tilapia. Similarly, control of cyp19a1a expression may be relevant for sex reversal of females into males, given the aforementioned differential growth between sexes. Recently, Wang et al. (2016), using ovaries and testes of young Nile tilapia, showed that miR-17-5p and miR-20a were highly expressed in the ovaries and negatively regulated DMRT1 expression, suggesting that these miRNAs could induce estrogen production by inhibiting DMRT1 expression and promoting cyp19a1a expression in Nile tilapia. They found that miR-138, miR-338, and miR-200a negatively regulated cyp17a2 (Wang et al., 2016), which is involved in 20b-dihydroxy-4-pregnen-3-one biosynthesis and thus might be essential for spermatogonial cell proliferation and spermatogenesis (Eshel et al., 2014). Wang et al. (2016) also showed that the miRNAs miR-456 and miR-138 negatively regulate AMH and that lower expression of these miRNAs promotes testis differentiation by allowing AMH to be expressed in testis.

Another aspect to be investigated is immunity. Infected animals cause economic losses and risks to human health. *Streptococcus iniae* causes high mortality and huge economic losses in tilapia cultures in China. This outbreak has seriously hindered the development of the tilapia industry (Qiang et al., 2017a). Qiang et al. (2017a) found that miR-92d regulates the expression of complement C3, which is the central component of the immune system. Furthermore, Qiang et al. (2017b) found three miRNAs (miR-310, miR-92, and miR-127) that were upregulated and four (miR-92d, miR-375, miR-146, and miR-694) that were downregulated by comparing a control group of Nile tilapia with a group infected with *Streptococcus iniae*. In another study of infection by Streptococcus, but using *Streptococcus agalactiae*, Wang et al. (2016) applied high-throughput sequencing followed by functional enrichment and found1121 differentially expressed miRNAs that target 41961 genes during infection. These analyses provide data for future studies of host-pathogen interactions among Nile tilapia and *S agalactiae*. In addition, Wang B. et al. (2017) identified 1981 miRNAs involved in the immune response against meningo encephalitis caused by *Streptococcus agalactiae* in Nile tilapia. Although these data are important, the studies of Wang et al. (2016) and Wang B. et al. (2017) are deficient because of their failure to find miRNA biomarkers that are more effective or that can be manipulated.

#### Atlantic salmon (*Salmo salar*, salmonidae)

Atlantic salmon is a domesticated fish of notable economic interest for wild fisheries and aquaculture production. Since 1970, Atlantic salmon have been intensively selected for genetic traits to improve growth performance, considerably benefiting aquaculture (Bentsen and Thodesen, 2005). The increased growth of this species is estimated at ~14% per generation (Gjedrem, 2010). Genome knowledge of salmonids is advanced in comparison to other farmed fish species (Bekaert et al., 2013); however, there are limited studies describing salmonid miRNAs.

To provide better tools for future analysis of miRNAs, Zavala et al. (2017) identified and validated appropriate endogenous reference miRNA genes. Zavala et al. (2017) showed that the ssa-miR-99-5p gene was the most stable overall and that ssamiR-99-5p and ssa-miR-23a-5p were the best combination.

The first screen for salmonid miRNAs was performed by Barozai (2012) using an *in silico* approach to predict miRNAs in salmon genomes. They detected let-7a-3p as a regulator of zonadhesin-like and growth hormone 2 gene, miR-142-5p as a regulator of heparin-binding growth factor 1, and miR-144 as a regulator of the growth factor receptor-bound protein 2. They also found that miR-430 regulates the transforming growth factor-beta-induced protein ig-h3, miR-451 blocks the anti-dorsalizing morphogenic protein, and miR-1594 activates both titin-cap (telethonin)-like mRNA and growth hormone receptor isoform 2. All of these miRNAs and targets are associated with growth and developmental processes.

Andreassen et al. (2013) characterized miRNA genes in Atlantic salmon by performing deep sequencing analysis of small RNA libraries from nine different tissues and revealed a total of 180 evolutionarily conserved mature miRNAs and 13 distinct novel mature miRNAs. Later, Bekaert et al. (2013) deep sequenced miRNA libraries of young juveniles (4 months old) and identified 547 miRNA transcripts that mapped to 88 miRNA distinct genes.

Johansen and Andreassen (2014) validated miR-25-3p and miR-455-5p as the best-performing two-reference gene combination suitable for quantitative expression analysis in Atlantic salmon. Such findings are relevant for aquaculture because these two miRNA reference genes are now useful for appropriate diagnostics tests, such detection of infection by viral RNA through expression. In addition, Kure et al. (2013) identified the differential expression of miRNAs subjected to an acid environment through RNA-Seq. They found 4 down- and 14 upregulated miRNAs between exposed groups and the control, suggesting alterations in a number of physiological responses that ultimately may interfere in animal growth performance.

The modulatory effect of miRNAs in collagen formation in salmon muscle has also been investigated (Mitchie, 2001). Muscle firmness is appreciated by consumers, and inadequate processing of salmon meat reduces firmness in selected adult salmons (Moreno et al., 2016). miR-29a, which is highly conserved between *Salmo salar* and *Danio rerio* (Andreassen et al., 2013), is the major factor in collagen formation and showed low expression in human fibroblasts with systemic sclerosis (Maurer et al., 2010). Thus, miR-29a may be a future target in expression studies to improve filet quality with a high potential for applications in aquaculture.

Furthermore, in salmon aquaculture, precocious puberty creates welfare problems and consequently inflicts economic damage. Skaftnesmo et al. (2017) explored which miRNAs regulate mRNAs during initiation of puberty, and several regulated miRNAs in the pubertal stage had earlier been associated (miR-20a, miR-25, miR-181a, miR-202, let7c/d/a, miR-125b, miR-222a/b, miR-190a) or have now been found connected (miR-2188, miR-144, miR-731, miR-8157) to the initiation of puberty.

In addition, there have been some studies on miRNAs associated with immunity. Andreassen et al. (2017) identified miRNAs responding to salmonid alphavirus (SAV) at different time points post-infection. They identified 20 differentially expressed miRNAs that may be important in viral-host interactions in Atlantic salmon. Following the same field of interest, Valenzuela-Miranda et al. (2017) studied the expression of miRNAs during infection with *Piscirickettsia salmonis* in the head, kidney and spleen. *Piscirickettsia salmonis*, a facultative intracellular bacterium, causes salmonid rickettsial septicemia (SRS), which is associated with major mortality in salmonid aquaculture (Mauel and Miller, 2002). The miRNA families miR-181, miR-143, and miR-21 were the most abundant in control groups, while miR-21, miR-181, and miR-30 were the most abundant in animals infected with *P. salmonis* (Valenzuela-Miranda et al., 2017). Sea louse *Caligus rogercresseyi*, which affects Chilean aquaculture, were studied during infestation in Atlantic salmon and the most abundant families were mir-10, mir-21, mir-30, mir-181, and let7 in skin, head and kidney (Valenzuela-Muñoz et al., 2017).

Overall, the collection of experimentally identified Atlantic salmon miRNAs provide an important resource for functional genome research in Neotropical fish and, in particular, helps to determine the actual contribution of miRNAs to phenotypic variation in economic and biologically fundamental characteristics.

#### Rainbow trout (*Oncorhynchus mykiss*, salmonidae)

Rainbow trout, the most widely cultivated cold freshwater fish in the world, has been improved in terms of its growth and development by animal breeding programs globally (Sae-Lim et al., 2013). This species is also a model organism for genome-related research on comparative immunology, carcinogenesis, toxicology, disease ecology, physiology, evolutionary genetics, and nutrition (Thorgaard et al., 2002).

Juanchich et al. (2016) provided a repertoire of 2946 miRNA loci in the rainbow trout genome, including 445 already known, in 38 different samples corresponding to 16 different tissues. These data represent the first characterization from a wide variety of tissues and provide a novel resource for research. Subsequently, Mennigen and Zhang (2016) created and reported the microtrout, a comprehensive database, which was developed to implement an algorithm to predict relationships among miRNA-mRNA targets.

Ramachandra et al. (2008) discovered 14 miRNAs in early embryos (5 dpf); among these, miR-21, miR-30d, miR-92a, miR-200, and miR-26 are associated with differentiation and development. Salem et al. (2010) showed that miR-133, which is known to be enriched in mammalian muscle (Shingara et al., 2005; Ason et al., 2006; Chen et al., 2006), is also highly expressed in trout skeletal muscle and therefore is of interest in animal breeding.

Other miRNAs involved in growth and nutritional support metabolic pathways have also been reported. A pioneer study by Mennigen et al. (2012) describes the post-prandial regulation of lipid and glucose metabolism by miRNAs. Among these miRNAs, they predicted conserved targets in fish and humans for miR-103, miR-107, and miR-143 homologs. Research has also shown miR-33 and miR-122b were upregulated, whereas miR-122a was downregulated. MiR-33 and miR-122b act on the hepatic insulin pathway to stimulate lipogenesis and inhibit lipolysis (Fernández-Hernando et al., 2011). MiR-33 and miR-122b may promote lipogenesis and simultaneously inhibit lipolysis. The inhibition of miR-33 expression in mice (*Mus musculus*) resulted in a low level of VLDL triglyceride (Fernández-Hernando et al., 2011). Similarly, inhibition of miR-122 resulted in increased fatty acid oxidation and decreased fatty acid synthesis rates (Krützfeldt et al., 2005; Esau et al., 2006; Elmén et al., 2008). MiR-122b was inhibited alongside an increase in plasma triglycerides, a final product of the lipogenic pathway (Li et al., 2009; Mennigen et al., 2012). Mennigen et al. (2013) analyzed the expression of miRNAs by quantitative real-time RT-PCR in rainbow trout fingerlings while switching from endogenous to exogenous feeding and showed a decrease in miRNA-33 and miRNA-122a/b isomiRs.

Paneru et al. (2017) aimed to investigate the association of miRNA expression with muscle growth and the effects on growth of single nucleotide polymorphisms (SNPs) in miRNA binding sites. They found 90 miRNAs that showed differential expression and were strongly associated with phenotypic variations. Among the 204 SNPs with 3′ UTRs targeted by miRNAs, 78 SNPs may be associated with changes in 5 muscle traits. The expression pattern of 12 miRNAs, including mir-1, mir-133 and mir-206, was validated by real time PCR.

On the basis of these studies, it is possible that down regulation of the above miRNAs could contribute to weight gain in rainbow trout. Therefore, miRNAs could possibly be experimentally manipulated in live animals to improve the production indices in rainbow trout.

#### Grass carp (*Ctenopharyngodon idella*, cyprinidae)

Grass carp is extensively cultivated in eastern Asia and is one of the most important freshwater fish globally (Liu et al., 2009). Grass carp was introduced into 115 countries worldwide, and in at least 58 of these (~50%) it appears to have self-sustaining populations (Fishbase, 2015) and high performance.

Zhu et al. (2014) reported that several miRNAs are likely involved in fast-twitch skeletal muscle growth in grass carp based on their analysis of the response to rapid refeeding following fasting. They recorded changes in the expression levels of eight miRNAs (miR-1a, miR-181a, miR-133a, miR-214, miR-133b, miR-206, miR-146, and miR-26a) shown to be involved in a strong resumption of myogenesis (Zhu et al., 2014).

Xu et al. (2014) studied miRNA expression stability in embryos at distinct developmental stages as well as in several tissues from adults in an effort to establish the best reference genes for quantitative expression analysis. Seven miRNAs (miR-126-3p, miR-101a, miR-451, miR-22a, miR-146, miR-142a-5p, and miR-192) were found to have optimal stability and should be individually prioritized according to the stage and tissue of interest.

Analyzing two grass carp lineages, *Aeromonas* hydrophila-susceptible (SGC) and -resistant (RGC), Xu et al. (2015) found miRNAs related to the immune system. MiR-118 and let-7i are differentially expressed and target both tlr4 and nfil3-6 genes. Let-7i is predominantly expressed in the spleen during bacterial infection and displays a noticeable difference in expression between SGC and RGC (Xu et al., 2015). The let-7 family is known to regulate multiple genes related to the cell cycle and proliferation (Yang et al., 2008), and let-7i was shown to influence innate immunity (Chen et al., 2007). Recently, Xu et al. (2016) studied these two carp lineages using two kidney miRNA transcriptomes from SGC or RGC infected with the highly pathogenic A. hydrophila and identified nine miRNAs differentially expressed between these groups. Furthermore, the spatial and temporal expression of a novel miRNA (cid-miRn-115) and miR-142a-3p suggests that they are potential regulators of anti-bacterial activity (Xu et al., 2016). Overexpression of these miRNAs resulted in a visible change in the immune effector activity in *C. idella* kidney cells, and bioinformatics analysis shows that they directly regulate tlr5 expression (Xu et al., 2016), which is associated with the innate immune response (Yoon et al., 2012).

Therefore, all these miRNAs interfere with grass carp health and should be further investigated for improving fish resistance to diseases that account for great economic losses in aquaculture.

## Discussion

### Biological implications of miRNA-mediated regulation

In fish, numerous miRNAs have been continuously identified, although only a small fraction have known functions (Figure 1; Table 2). Regarding Neotropical fish, these numbers are even smaller since very few species have been studied regarding miRNA functions (all species covered in this review). Considering that miRNAs have an important function in shaping both morphological and physiological phenotypes, and its function is highly conserved within vertebrates, analysis of miRNA profiles from other species might potentially be useful to understand and to provide wide applications in Neotropical fish breeding.

**Table 2:** Summary of miRNA associated functions described in each species reviewed in the manuscript.

**Group**	**Species**	**miRNA**	**Associated function**
Neotropical	Tambaqui	miR-122	Regulation of cholesterol metabolism
		miR-1; miR-206	Myoblast differentiation
	Pacu	miR-133a/b	Myoblast proliferation
Others	Nile tilapia	miR-499	Differentiation of slow-twitch muscle fibers
		miR-1; miR-206	Myoblast differentiation
		miR-133a/b	Myoblast proliferation
		miR-30c	Regulation of salt tolerance
		miR-429	Regulation of osmotic stress
		miR-21	Regulation of alkaline tolerance
		miR-122	Regulation of stress response
		miR-29; miR-129	Overexpressed in Ovary
		miR-129	Ovary maturation
		miR-29	Oogenesis
		miR-33a; miR-132; miR-135b; miR-212	Overexpressed in Testis
		miR-33a	Testis maturation
		miR-4585	Sex dettermination
		miR-17; miR-20a	Regulation of estrogen production
		miR-138; miR-338; miR-200a	Spermatogonial cell proliferation and spermatogenesis
		miR-456; miR-138	Testis differentiation
		miR-92d	Regulation of C3 complement expression
		miR-310, miR-92, and miR-127	Overexpressed in *Streptococcus agalactiae* infection
		miR-92d, miR-375, miR-146, and miR-694	Downregulated in *Streptococcus agalactiae* infection
	Atlantic Salmon	let-7a; miR-142; miR-144; miR-430; miR-451; miR-1594	Regulation of growth
		miR-29a	Collagen formation
		miR-20a; miR-25; miR-181a; miR-202; let7c/d/a; miR-125b; miR-222a/b; miR337 190a; miR-2188; miR-144; miR-731; miR-8157	Initiation of puberty
		mir-10, mir-21, mir-30, mir-181 and let7	Overexpressed in *Caligus rogercresseyi infection*
		miR-21, miR-181 and miR-30	Overexpressed in *Piscirickettsia salmonis* infection
	Rainbow trout	miR-21, miR-30d, miR-92a, miR-200, and miR-26	Cell differentiation and embryo development
		miR-103, miR-107, and miR-143	Post-prandial regulation of lipid and glucose metabolism
		miR-33; miR-122b	Regulation of lipogenesis and lipolysis
	Grass carp	miR-1a, miR-181a, miR-133a, miR-214, miR-133b, miR-206, miR-146, and miR-26a	Fast-twitch skeletal muscle growth

Although limited functional studies on farmed fish miRNAs have been conducted, they have provided sufficient evidence for linking miRNA activity to biological aspects of economic interest, such as the control of muscle development and hypertrophy, regulation of oocyte and testis maturation, sex differentiation and physiological resilience to diseases and adaptation to environmental changes. Additionally, deep exploration of classic genetic aspects of miRNA function, such as miRNA mapping, control of expression and genomic context, have contributed to understanding the genetic basis of miRNA expression and have led to new insights into the influence of miRNA on an assortment of complex traits.

Since interactions between miRNAs and their targets can be strongly conserved among species, several interactions verified in previous studies could be extrapolated to Neotropical fish species, thereby remarkably improving fish aquaculture. For instance, the role ofmiR-1, miR-206, and miR-133 during myoblast proliferation and differentiation is recognized to interfere in the hypertrophic growth of skeletal muscle. These interactions has been shown in Nile tilapia (Nachtigall et al., 2015) and may potentially be a widely conserved miRNA-target interaction modulating growth of several Neotropical fishes, as already reported for pacu (Duran et al., 2015; Paula et al., 2017). In the skeletal muscle, the main edible part of the fish, miR-1, miR-133a, and miR-206 have conserved expression patterns in all farmed fish species, which makes them interesting molecules for modulating muscle development and growth. Particularly, in Neotropical fishes, the study of myomiRs (i.e., miRNAs specifically or enriched in this tissue) showed important data that can bring advances to increase their production to its full potential. Moreover, the fact that these miRNAs were found to play the same functional roles in the skeletal muscle of several other animal species corroborates hypotheses about their pivotal importance in gene regulatory networks.

For the large number of non-model fishes that do not have their complete genome sequenced or for those for which little is known about their genome and its interactions, advanced computational tools are becoming sufficiently capable and precise to achieve good and true assessments of the data and genome from model fish species that are evolutionarily close to non-model fish. Consequently, there is less of a gap or challenge between genomic information of non-model and model fishes. Some muscle miRNAs have well-defined target genes, as miR-206 regulates IGF-1; miR-1, miR-122, and miR-462 control IGF-2a; the let-7 family regulates MSTN; miR-103 and miR-107 modulate GHR and FSHR; and miR-138 and miR-211 control LHR. All these genes targeted by muscle miRNAs can be experimentally overexpressed or under expressed through molecular techniques aiming to obtain fishes carrying desired phenotypes. Furthermore, some fish miRNAs have well-defined conserved functions also occurring in humans and mice, and could be controlled to enhance production. For example, miR-29a acts in collagen formation as has conserved targets among fish, mouse and human, implying that this miRNA is a good candidate to be modulated in Tambaqui with the goal of improving filet quality.

Other aspects where miRNAs stand out pertain to the control of fish disease responses that specifically affect confined shoals, owing to the appearance of various stressors, such as poor water quality, high density, and inadequate diets. The megalocytivirus, from the Iridoviridae family, for example, has been a focus of research because it usually causes significant mortality to a wide range of hosts in aquaculture, leading to pronounced economic losses (Subramaniam et al., 2012). For instance, Nile tilapia (Subramaniam et al., 2016), Atlantic salmon (Crane and Hyatt, 2011), rainbow trout (Ariel and Jensen, 2009; Crane and Hyatt, 2011) and grass carp (Subramaniam et al., 2012) are confirmed hosts of megalocytivirus. Furthermore, grass carp is a potential carrier of megalocytivirus, but infection does not unavoidably cause mortality or clinical changes in this species (Subramaniam et al., 2012). Thus, studies could examine whether miRNAs differentially expressed would play a role in maintaining grass carp resilience to megalocytivirus infection and symptoms, and subsequently to test for a prospective application to Neotropical fish.

Another key aspect in fish biology pertains to the control of sexual differentiation and maturation. Experiments on Nile tilapia have shown that miR-456 and miR-138 inhibit testis differentiation by targeting the Amh gene (Wang et al., 2016), and the high expression of miR-4585 in males interferes with the sexual reversal mechanism. Together, these miRNAs can be good biomarkers for investigating both the mechanisms influenced by sex and mechanisms that influence sexual development, many of which are important parameters for efficient production. For example, some of these findings could be further investigated to produce monosex populations of species that females grows larger than males, such as tambaqui. Overall, this area is still in its infancy, and additional studies focusing on miRNA pathways related to sexual development in fishes will be of great value for improving fish breeding.

Environmental temperature plays a key role in maintaining the life cycle of any fish species. Understanding the molecular mechanisms involved in acclimatization at different temperatures is fundamental in the current context because of global warming. In addition, these molecular mechanisms can be used to genetically select fish in aquaculture programs. For example, the cultivation of tilapias and black pacu is restricted to tropical and subtropical areas, since they prefer temperatures between 27 and 32°C. Handling and transport at low temperatures (< 22°C), especially after winter, leads to severe reduction of appetite and increased risk of disease. Furthermore, the culture of these fish at a temperature below 14°C is generally lethal. As a result, tilapia and black pacu breeding companies cannot expand their activities to low-temperature locations. Therefore, miRNAs have been proposed as molecular markers to select cold-tolerant fish. In zebrafish, 25 differentially expressed miRNAs were identified in individuals cultured for 10 days at a temperature of 10°C, which is related to a cellular response of adaptability to the environment (Yang et al., 2011). Thus, miRNAs could be studied as epigenetic markers for the selection of cold tolerant Neotropical fish, allowing the production of several species in subtropical and temperate regions.

Regarding general metabolism, miRNAs that regulate lipid and glucose metabolism are highly conserved between humans and fishes. For instance, miR-103, miR-107, miR-122, and miR-143 have constrained metabolic functions in vertebrates and can be targeted in future genetic therapies. Similarly, salt tolerance is an interesting trait and Nile tilapia has strong tolerance associated with the expression of miR-30c and miR-429, which can be broadly investigated and modulated in less tolerant Neotropical species to increase their productivity.

One of the major challenges, if not the greatest, for the use of miRNAs to increase the production of farm animals is the control of miRNA expression and consequently the control of genes and their products. Such control would allow researchers and farmers to have absolute control over a range of characteristics that would make the animals more productive or exhibit better quality. Efforts have been made to this end, and in the future, it is likely that miRNAs can be fully manipulated. However, in a genomic context, miRNAs can already be very well-exploited with the purpose of improving animal production.

### Biological implications of miRNA genomic context and regulation of expression of complex traits

Characterization of miRNA signatures of expression for quantitative trait loci (named “miR-eQTL”) can produce insights into regulatory mechanisms of miRNA transcription and assist in clarifying the role of miRNAs as orchestrators of complex biological traits.

Experiments detecting the quantitative trait locus (QTL) related to important breeding traits have uncovered diverse molecular markers useful for fish production (Sonesson, 2007; Canario et al., 2008). Recently, the relationship between miRNAs and QTLs has been found to interfere in a myriad of processes, as in sexual differentiation and determination in Nile tilapia (Eshel et al., 2014) and in immune defense against viral infection in Atlantic salmon (Lowe et al., 2014). Additionally, colligated assessment of miRNA signatures and SNP within QTLs has contributed to the recognition of several miRNA regulatory loci of interest. Such miR-eQTLs can be used to determine phenotypic information through the integration of QTL polymorphisms with transcriptome data, including genomic loci of miRNA that contribute to the variation of mRNA expression levels. An analysis in European seabass, another economic relevant species, identified 20,779 SNPs over 1,469 gene loci and intergenic spacers (Kuhl et al., 2011), showing that they can be extensively used as genetic markers in population and molecular studies.

The integration of QTL and miRNA allowed the identification of key regulatory pathways involved in human liver diseases (Gamazon et al., 2013). This approach could be used in fish to assess susceptibility to diseases and other biological aspects, such as muscle development. Indeed, miR-eQTLs are promising molecular tools for enhancing aquaculture productivity and/or species conservation. Similarly, global identification and characterization of miRNAs and QTLs could be an important source for further understanding how coding and non-coding DNA work together to generate attractive phenotypic traits for selection programs (Cerdà and Manchado, 2013).

Although miRNAs have the potential to be used as molecular markers in fish genetic breeding programs, some limitations persist in their broad application in aquaculture. For instance, the assessment of miRNA expression levels is usually performed in specific tissues, requiring euthanasia of the fish, which would make it impossible to use the animals for subsequent selection programs. Another drawback comes from the presence of miRNAs in biofluids, such as blood and urine (Harrill et al., 2016) that were not directly expressed in these fluids, but rather resulted from leakage owing to cell injury, cell death, or active secretion during manipulation. As an alternative to euthanasia for tissue collection, evaluation of circulating miRNA levels in body fluids can be performed to monitor fish metabolism, allowing animals to be further used for genetic selection. In this way, miRNAs could be evaluated in fish blood, since circulating miRNAs are already being proposed as stable epigenetic biomarkers in several mammals, including humans (Harrill et al., 2016; Kasimanickam and Kastelic, 2016).

Another aspect inherent in miRNA modulation pertains to the characteristics of multigenic regulation, that is, a single miRNA can regulate several genes in different biological contexts. So when miRNA expression is altered artificially, the expression of several target genes can be altered. This is one of the challenges of manipulating miRNAs that makes it difficult to obtain the desired final phenotype. This challenge can be overcome or minimized by controlling the modulation of miRNAs specifically or highly expressed in certain tissues during temporal windows of development. For this approach, it is necessary to validate the real interactions between target miRNAs (Box 2) in tissues and various cell types. It will then be possible to reduce or eliminate off-target effects and obtain the desired benefits on the stock. The problem of small size and low abundance of some miRNAs has been circumvented through the development and implementation of last generation sequencing techniques and advanced bioinformatics tools.

In addition, to evaluate whether miRNAs with described functions in one species can change the phenotype of other organisms, genetic reverse techniques for gene knock in or knockout can be performed. This approach works by changing gene expression to rigorously test its function, helping to determine the potentially beneficial aspects of down and up regulation in farmed fish species. Additionally, when these effects are confirmed as molecularly conserved in several species, genome-editing analysis can be broadly performed to modulate expression levels of one or a subset of miRNAs in related and non-related fish species. Cutting-edge genome editing techniques have been recently improved with the CRISPR-Cas system and could positively be implemented for aquaculture enhancement. Several papers have already suggested that CRISPR-Cas studies could be widely applied in fish, as first proven by studies using zebrafish models (Bassett et al., 2014).

Based on the aforementioned discussion, we envision that future research on farmed Neotropical fishes will greatly benefit from the ongoing comparative analysis of transcriptomes, which will provide the roadmap for the development of practical applications to expand animal breeding programs. Certainly, studies on farmed fishes that focus on the identification of interactions between SNPs and miRNAs are another important direction. SNPs in miRNA binding sites or in the miRNA precursor sequences may largely impact the desired phenotype and provide the best use of genomics and bioinformatics in animal breeding and aquaculture. Degradome sequencing technology and miRNA-seq can be applied to populations rather than to a few individuals to assess SNPs associated with specific desired phenotypes. This feature could provide an immediate tool for selecting both larval and adult fishes carrying superior traits of economic interest and improve profits.

Finally, gene expression regulation in its various forms is considered one of the fastest and most effective mechanisms underlying adaptive evolution. Thus, several years ago, gene regulation was recognized as a powerful force for diversification and speciation. As was well-discussed in this paper, miRNAs are key molecules involved in the regulation of genes in all complex organisms, including phenotypic characteristics that confer better production or selective advantages in nature. In addition, Franchini et al. (2016) have shown that several species of Neotropical fishes, given their evolutionary history that forces them to form small populations due to physical environmental changes, are and will still serve as good biological models in studies involving molecular mechanisms associated with speciation caused by changes in gene expression. Consequently, miRNAs play a fundamental role in this process. Thus, these species may be at the forefront of studies involving miRNAs and gene regulation as speciation mechanisms.

## Conclusions and perspectives

In conclusion, this review on fish miRNAs shows that these small molecules are great targets for understanding Neotropical fish biology and that miRNAs possess very attractive features for their immediate implementation as biomarkers that can be used to select adaptive traits for a specific environment in worldwide aquaculture. The existence of highly conserved miRNAs among vertebrates is relevant and may lead to the broad application of knowledge acquired from one fish species to another or even allow for genome editing technology transferability within distinct vertebrate groups. Many miRNA-mediated biological characteristics are available for study and implementation into Neotropical fish farming in distinct captivity environments. Genome editing is the best approach to increase or reduce miRNA expression and could contribute to the improvement of important characteristics that can enhance global aquaculture production.

## Author contributions

Literature survey: MH, AO, PN, JC. Data discussion: MH, AO, PN, JC, VC, AH, DP. Writing of the first draft of the manuscript: MH, AO, PN, JC. Critically revision and final manuscript writing: MH, VC, AH, DP. All authors read and approved the final manuscript.

### Conflict of interest statement

The authors declare that the research was conducted in the absence of any commercial or financial relationships that could be construed as a potential conflict of interest. The reviewer DTH declared a shared affiliation, with no collaboration, with several of the authors (MH, AO, PN, JC, and DP) and to the handling Editor.
